# Obesity Increases Airway Hyperresponsiveness via the TNF-α Pathway and Treating Obesity Induces Recovery

**DOI:** 10.1371/journal.pone.0116540

**Published:** 2015-02-06

**Authors:** Joo Young Kim, Jung-Ho Sohn, Jae-Hyun Lee, Jung-Won Park

**Affiliations:** 1 Ewha Womans University College of Pharmacy, Research Institute of Pharmaceutical Sciences, Seoul, South Korea; 2 Department of Internal Medicine, Institute of Allergy, Yonsei University College of Medicine, Seoul, South Korea; Leiden University Medical Center, NETHERLANDS

## Abstract

Obesity is a known risk factor for allergic asthma. It has been recognized as a key player in the pathogenesis of several inflammatory disorders via activation of macrophages, which is also vital to the development of allergic asthma. We investigated the mechanism of obesity-related asthma and whether treating obesity through exercise or diet ameliorates the severity of asthma in the obesity-related asthma model. We generated diet-induced obesity (DIO) in C57BL/6 mice by high-fat-feeding and ovalbumin-induced asthma (lean-OVA or DIO-OVA). The DIO-OVA mice were then treated with tumor necrosis factor (TNF)-α neutralizing antibody as a TNF-α blockade or a Cl_2_MDP-containing liposome to induce an alveolar macrophage deficiency. To treat obesity, the DIO-OVA mice were under dietary restrictions or exercised. The pathophysiological and immunological responses were analyzed. Airway hyperresponsiveness (AHR), serum IgE and TNF-α levels in the lung tissue increased in the DIO-OVA mice compared to the lean-OVA mice. Both the TNF-α blockade and depletion of alveolar macrophages in the DIO-OVA mice decreased AHR compared to the DIO-OVA mice. Treating obesity by exercise or through dietary means also reduced pulmonary TNF-α levels and AHR in the DIO-OVA mice. These results suggest that restoring normal body weight is an appropriate strategy for reducing TNF-α levels, and controlling inflammation may help improve asthma severity and control in obesity-related asthma.

## Introduction

Obesity is a metabolic disease and a major risk factor for several noncommunicable diseases, such as diabetes, and cardiovascular diseases. The World Health Organization estimates that more than 1.4 billion adults are overweight, and of these overweight adults, over 200 million men and nearly 300 million women are obese [1]. Obesity is also associated with a later onset of asthma in the development, control and severity [2]. Recently, several studies have focused on the heterogeneity of asthma phenotypes based on clinical characteristics, triggers, or general inflammatory processes, even though asthma has been considered a single disease for years [3]. Obesity may not be only associated with lung mechanics, such as airway closure during tidal breathing and reduced expiratory residual capacity [4], but also with a high expression of certain inflammatory mediators, such as tumor necrosis factor (TNF)-α, interleukin (IL)-6, and leptin [5, 6].

The mechanisms of action between obesity and asthma are not fully understood. Clinical studies showed that subjects with obesity-related asthma usually have noneosinophilic asthma, unexplained by Th2 immune responses [2, 7]. In addition to the physiologic effects of obesity on lung function, several investigators have hypothesized that obesity leads to a state of low-grade systemic inflammation that may act on the lungs to exacerbate asthma [8]. TNF-α is an important proinflammatory cytokine and has been implicated in the mechanisms of several inflammatory diseases, such as allergic asthma, inflammatory bowel disease, and rheumatoid arthritis [9]. As a common factor in asthma and obesity, TNF-α might be an important target for treating obesity-related asthma [10].

To treat allergic symptoms in obesity-related asthma, several investigators have suggested that weight reduction by diet, exercise, or bariatric surgery might prevent the development of asthma, or at least decrease asthma-related symptoms, and improve asthma-specific quality of life, as measured by questionnaire or degree of health care utilization [11–13].

In this study, we investigated the mechanism of obesity-related asthma and whether treating obesity through weight reduction affects the pathogenesis of the obesity-related asthma model.

## Materials and Methods

### Animals

Female C57BL/6 mice (4-weeks old) were purchased from Japan-SLC (Hamamatsu, Japan) and were randomly allocated to experimental groups. Total of three independent experiments were performed and each experimental data was obtained from five mice per group. According to the retrospective statistical calculation for this study (http://www.biomath.info/power/index.htm), more than five mice per each group would be needed to yield a power of 80% (assuming α, 2-tailed, was set at 0.05). This study was approved by the Institutional Animal Care and Use Committee (2010–0223), Yonsei University College of Medicine (Seoul, Republic of Korea), which has been fully accredited by the Association for Assessment and Accreditation of Laboratory Animal Care International. This study adhere to the ARRIVE Guidelines for reporting animal research (S1 ARRIVE Checklist).

### Diet-induced obesity

**Figure 1:**
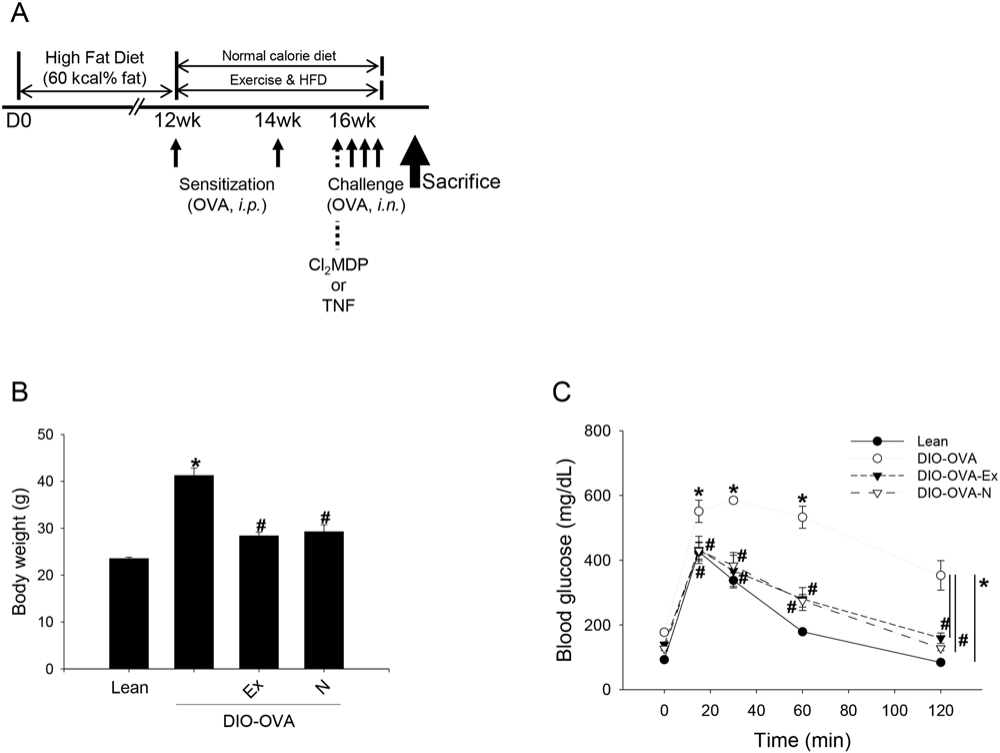
Mouse models in this study. (a) Scheme of this study. C57BL/6 mice fed HFD for 16 weeks and some of the DIO mice underwent OVA sensitization and challenge (DIO-OVA). Some of the DIO-OVA mice were treated with TNF-α neutralizing antibody for TNF-α blockade or a Cl_2_MDP-containing liposome for alveolar macrophage depletion. For the treatment of obesity, the DIO-OVA mice performed voluntary exercise (DIO-OVA-Ex) or underwent dietary restriction (DIO-OVA-N) after 12 weeks of HFD feeding. (b) Body weight and (c) blood glucose tolerance was measured at the end of 16 weeks after HFD feeding. *, Statistical significance to lean mice (p<0.05); ^#^, Statistical significance to DIO mice. *i.p.*, intraperitoneal injection; *i.n.*, intranasal injection; *TNF*, TNF-α neutralizing antibody. Error bars indicated mean±SEM of five mice per group. All data are representative of three independent experiments.

To make diet-induced obesity (DIO) mice, the 4-week-old C57BL/6 mice were fed a high fat diet (HFD) for 16 weeks. The HFD (D12492; Research Diets, Inc., New Brunswick, NJ) contained 60% kcal from fat. The lean mice, as a control, were fed a normal chow diet (D12450B; Research Diets, Inc.) containing 10% kcal from fat (Fig. 1A).

### Glucose tolerance test

A glucose tolerance test (GTT) was performed 16 weeks after HFD feeding. Mice were fasted overnight and were intraperitoneally injected with glucose (2 g/kg body weight; Sigma-Aldrich, St. Louis, MO). Blood from the tail vein was collected at 0, 15, 30, 60, and 120 min after glucose injection. Blood glucose levels were measured with a blood glucose meter (One Touch Ultra; Life Scan, Inc., Milpitas, CA).

### Voluntary running exercise

After 12 weeks of HFD feeding, some of the DIO mice were housed individually and performed voluntary exercise using a Mouse Igloo Fast-Trac (Bio-Serv, Frenchtown, NJ) for 4 weeks (Fig. 1A). They were monitored in the last week of exposure to the running wheels. To calculate exercise volume, we installed a counter on the wheel and converted the wheel RPM into a daily distance.

### OVA-asthma model

To make an established asthma model in lean or DIO mice, some of the mice underwent intraperitoneal sensitization with a mixture of OVA (100 μg per mouse; Sigma-Aldrich) and Imject Alum (100 μL per mouse; Thermo Scientific, Rockford, IL) at 12 and 14 weeks, followed by an intranasal challenge 3 times with the same antigen (OVA, 10μg per mouse) via intranasal route at 16 weeks. Two days after last OVA challenge, we sacrificed the asthmatic mice for analysis.

### Depletion of alveolar macrophages

Liposomes encapsulated with dichloromethylene diphosphonic acid disodium salt (Cl_2_MDP, Sigma-Aldrich) were prepared as previously described [14]. A suspension of Cl_2_MDP-liposomes contained about 6 mg Cl_2_MDP per milliliter suspension. Cl_2_MDP-containing liposomes (300 µg/50 µL/mouse) were administered intranasally to obese or lean mice 12 hours before the OVA challenge to eliminate alveolar macrophages.

### TNF-α blockade

To block TNF-α, goat anti-mouse TNF-α polyclonal antibody (10 μg/mouse; R&D Systems, Inc., Minneapolis, MN) or goat IgG (10 μg/mouse, R&D Systems) was injected via intravein route 12 hours before the OVA challenge.

### Measurement of lung function

Lung functions were measured as previously described [15]. Mice were anesthetized (pentobarbital sodium, 50 mg/kg injected intraperitoneally), ventilated (FlexiVent 5.1: SCIREQ, Montreal, Canada) and challenged with aerosolized-saline followed by an increasing dose of methacholine (MCh, Sigma-Aldrich). Aerosols were generated with an ultrasonic nebulizer (Omron Healthcare, Kyoto, Japan) and delivered to the inspiratory line of the FlexiVent using a bias flow of medical air. Baseline lung functions (such as R_L_, C_dyn_, R_aw_, G and H values) were measured at the stage of aerosolized saline nebulizing. Total pulmonary resistance (R_L_) and dynamic compliance (C_dyn_) were obtained using the forced oscillation technique, and airway resistance (R_aw_), lung tissue damping (G) and elastance (H) were measured with a constant phase model [16].

### Bronchoalveolar lavage fluid and lung homogenate

To collect bronchoalveolar lavage (BAL) fluid, the lungs were lavaged with 1 mL Hank’s balanced salt solution via the tracheostomy tube. BAL cells were counted with a hemocytometer, smeared by cytocentrifugation (Cytospin3, Thermo, Billerica, MA) at 1000 rpm for 3 min, and then stained with a Hemacolor Staining Kit (Merck, Darmstadt, Germany). BAL cells from each group were counted and classified as macrophages, lymphocytes, neutrophils, or eosinophils. To minimize the effects of subjective bias in the classification of the BAL cells, blind outcome assessment was used.

For protein extraction, lung tissues were homogenized in 20 mL/g tissue protein extraction reagent (Thermo Fisher Scientific Inc., Rockford, IL) using a tissue homogenizer (Biospec Products, Bartlesville, OK). Homogenates were incubated at 4°C for 30 min and then centrifuged at 1000 × g for 10 min. Supernatants were collected, passed through a 0.45-micron filter (Gelman Sciences, Ann Arbor, MI), and then stored at -70°C for assessment of cytokine levels. The measured cytokine levels were normalized to lung tissue weight and expressed as ng per mL per lung tissue.

### Enzyme-linked immunosorbent assay

TNF-α, and total and specific IgE levels from lung homogenates or blood sera were measured by means of enzyme-linked immunosorbent assay (ELISA) with commercially available materials. Briefly, TNF-α and total IgE levels were assayed with Mouse TNF-α DuoSet (R&D Systems) and BD OptEIA Mouse IgE ELISA Set (BD Pharmingen, San Diego, CA), respectively. Specific IgE levels were measured by modified-sandwich ELISA [17]. Briefly, the plates were coated with a purified rat anti-mouse IgE, and sera were used as test samples. Biotinylated-OVA and peroxidase-conjugated streptavidin were reacted in order. The reactions were read at 450 nm.Leptin and adiponectin levels from lung homogenates or blood sera were measured by means of ELISA with commercially available materials, Mouse Leptin and adiponectin DuoSet (R&D Systems), respectively.

### Histologic analysis

Periodic Acid-Schiff (PAS) staining was performed in the formalin-fixed/paraffin-embedded lung tissues. Tissue sections were examined with an Olympus BX40 microscope in conjunction with an Olympus U-TV0.63XC digital camera (Olympus Corp., Melvile, NY). Images were acquired using DP Controller and Manager software (Olympus Corp.).

### Flowcytometric analysis

For assessments of cell phenotypes (CD11b^Int^F4/80^High^ cells) in the lung tissue, multicolor-flow cytometric analysis was performed (LSRII; BD Biosciences). The data were analyzed using FACSDiva (BD Biosciences) or FlowJo ver.7.6.2 (Three Star, Ashland, OR) and expressed as a percentage value.

### Statistical analysis

The data are expressed as mean±standard error (n = 5). Statistical analyses were performed using SPSS ver. 12.0 (Chicago, IL). Groups in the GTT and the methacholine challenge tests were compared using two-way ANOVA with Tukey post hoc analysis, and the others were compared using one-way ANOVA with Bonferroni post hoc analysis. All differences were considered significant at *p*<0.05.

## Results

### 1. Physical characteristics of obese mice

During the experimental period, the DIO mice were fed a HFD and their body weights significantly increased by 75% more than the lean mice at 16 weeks (Fig. 1A-B). To restore normal body weight, the DIO mice then consumed food in the normal calorie range (DIO-N) or performed voluntary running exercise (DIO-Ex) from the 12th to 16th week. The DIO-Ex mice ran about 6.25±0.41 km per day on average in the last week of exercise. Both the DIO-N and DIO-Ex groups significantly reduced their body weight to that of lean mice (Fig. 1B). Because it is a metabolic disease, obese subjects spontaneously have glucose intolerance in their blood stream, similar to in type II diabetes mellitus. The DIO mice had impaired glucose tolerance, however both the DIO-N and DIO-Ex mice revealed improvements in glucose tolerance similar to that of lean mice (Fig. 1C).

To determine whether dietary obesity affects baseline lung function in a mouse model, airway and lung parenchymal mechanics were measured. R_L_ was significantly greater in DIO mice than in lean mice, and the increased R_L_ of the DIO mice was decreased in the DIO-N mice with statistical significance (Table 1).

**Table 1:** Baseline lung function in obese mice.

**Group (n = 5)**	**R_L_ (cmH_2_O/mL/s)**	**C_dyn_ (mL/cmH_2_O)**	**R_aw_ (cmH_2_O/mL/s)**	**G (cmH_2_O/mL)**	**H (cmH_2_O/mL)**
Lean	0.64±0.10	0.043±0.007	0.31±0.06	4.57±0.71	19.58±1.84
DIO	1.00±0.09*	0.032±0.007	0.67±0.24	7.66±2.43	25.65±7.31
DIO-Ex	0.79±0.17	0.048±0.003	0.43±0.11	4.25±0.34	17.16±1.35
DIO-N	0.63±0.06^#^	0.05±0.001	0.33±0.07	4.18±0.14	17.94±1.70

Values are means±SD; R_L_, pulmonary resistance; C_dyn_, dynamic compliance; R_aw_, airway resistance; G, tissue damping; H, tissue elastance; DIO, diet induced obesity; Ex, free-wheel exercise; diet, normal chow diet; *, P<0.05 compared with control group; #, P<0.05 compared with DIO group. All data are representative of three independent experiments.

### 2. Obesity exacerbates allergic lesions in asthma-induced obese mice

**Figure 2:**
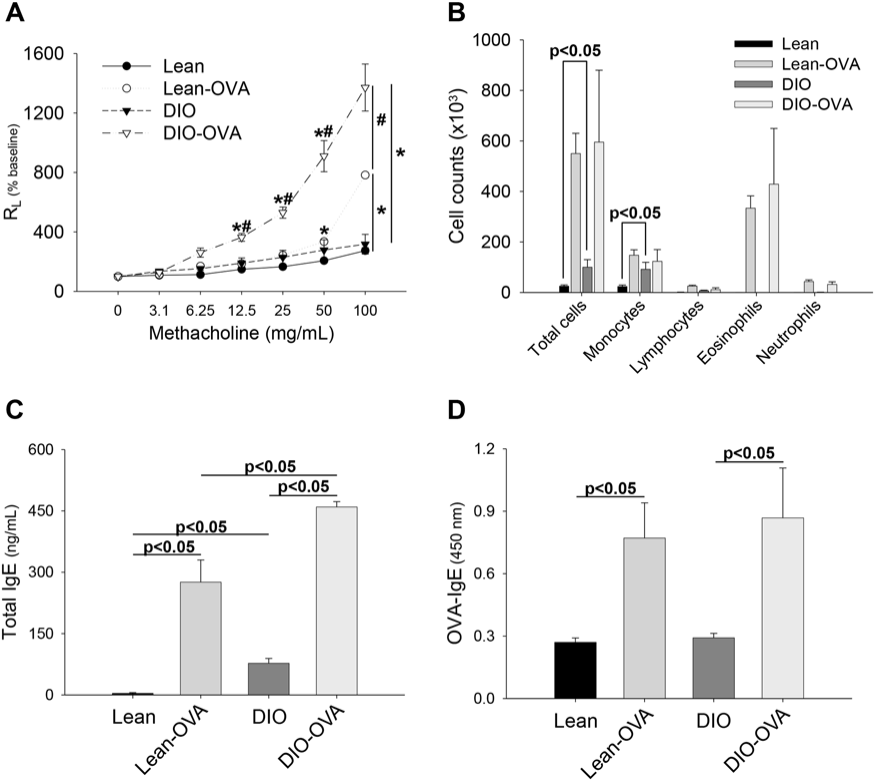
Obesity exacerbates asthmatic symptoms in the asthma model. (a) AHR, (b) inflammatory cell infiltrations in the BAL fluids, (c) total IgE and (d) OVA-IgE levels in the sera were measured in the asthma model (lean-OVA) and obesity-related asthma model (DIO-OVA). *, Statistical significance to their control group (lean or DIO; p<0.05); ^#^, Statistical significance between lean-OVA and DIO-OVA (p<0.05). Error bars indicated mean±SEM of five mice per group. All data are representative of three independent experiments.

To determine whether obesity exacerbates asthma, dietary-induced obese mice were subjected to a protocol of OVA sensitization and challenges (DIO-OVA mice; Fig. 1A). The DIO-OVA mice showed increased MCh airway hyperresponsiveness (AHR) compared with the asthma-induced lean (lean-OVA) mice (Fig. 2A). Inflammatory cell phenotypes in BAL fluid did not exhibit noticeable differences between the lean-OVA and DIO-OVA mice, but macrophages in the DIO mice were significantly increased compared with the lean mice (Fig. 2B).

As a notable feature of the asthma model, increased total and OVA-specific IgE levels in blood were revealed in both lean-OVA and DIO-OVA mice. However, there were no significant differences in OVA-specific IgE between the lean-OVA and DIO-OVA mice. Interestingly, specific IgE was not elevated, but total IgE levels in the DIO mice were significantly elevated compared with the lean mice (Fig. 2C-D).

As a representative pathologic change in the lung of the asthma model, goblet cell hyperplasia in the peri-bronchiolar area was measured, but we found no significant differences between the lean-OVA and DIO-OVA mice (S1 Fig.).

### 3. TNF-α aggravates pulmonary function in obesity-related asthma model

Previously, we proposed that TNF-α plays a key role in the allergic asthma model and alveolar macrophages can produce large amounts of TNF-α in the lung environment [15]. According to the increased presence of macrophages in the BAL fluid of DIO mice, we could expect that TNF-α plays an important role in the interaction between obesity and asthma.

**Figure 3:**
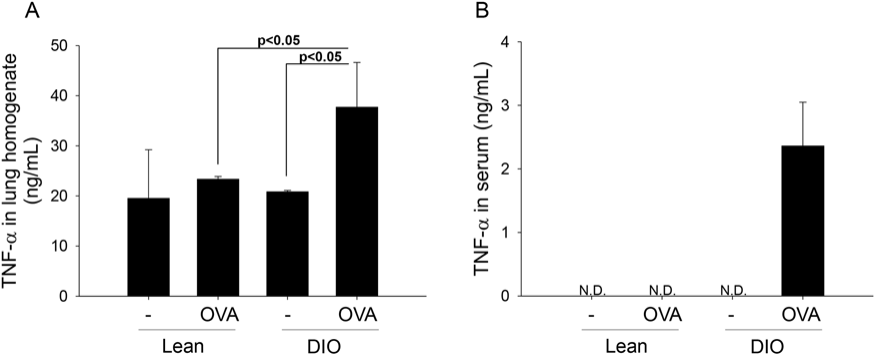
Obesity increases TNF-α levels in the asthma model. TNF-α levels in (a) the bronchoalveolar lavage fluids and (b) the blood sera were measured in the asthma models. The solid lines indicate statistical significance between each group (p<0.05). *N.D.*, not detected. Error bars indicated mean±SEM of five mice per group. All data are representative of three independent experiments.

We found significantly higher TNF-α levels in the lung homogenates of DIO-OVA mice compared with the lean-OVA mice (Fig. 3A). Furthermore, the DIO-OVA mice revealed markedly increased TNF-α levels in the sera (Fig. 3B).

**Figure 4:**
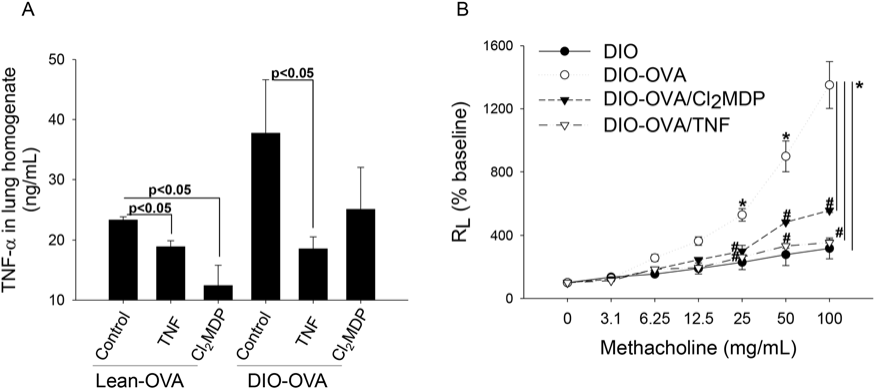
Depletion of TNF-α or alveolar macrophages attenuate lung dysfunction in the obesity-related asthma model. (a) Both lean-OVA and DIO-OVA mice were treated with TNF-α blockade antibody or Cl_2_MDP for depletion of TNF-α or alveolar macrophages, respectively, and TNF-α levels in the lung homogenates were measured. (b) MCh AHR was measured in the TNF-α or alveolar macrophage depleted DIO-OVA. *, Statistical significance to lean mice (p<0.05); ^#^, Statistical significance to DIO mice. *TNF*, TNF-α neutralizing antibody. Error bars indicated mean±SEM of five mice per group. All data are representative of three independent experiments.

To confirm the role of TNF-α in the obesity-related asthma model, TNF-α was blocked 12 hours before the first OVA challenge (Fig. 1A). The TNF-α blockade dramatically reduced TNF-α levels in the lung homogenates of the DIO-OVA mice (Fig. 4A), and improved AHR in both the lean-OVA (S2A Fig.) and DIO-OVA mice (Fig. 4B).

To examine the function of alveolar macrophages in the obesity-related asthma model, lean-OVA and DIO-OVA mice were treated with Cl_2_MDP-containing liposomes intranasally 12 hours before the first OVA challenge (Fig. 1A), and then alveolar macrophages were selectively eliminated (S2B Fig.). Cl_2_MDP reduced TNF-α secretion from the lung homogenates of lean-OVA and DIO-OVA mice (Fig. 4A), and attenuated AHR in both lean-OVA (S2A Fig.) and DIO-OVA mice (Fig. 4B) to the levels of control mice.

In this part, we found more decreased AHR in the TNF-α or alveolar macrophage depleted DIO-OVA mice compared with the TNF-α or alveolar macrophage depleted lean-OVA mice (Fig. 4B and S2A Fig.). Depletion of TNF-α or alveolar macrophages also reduced eosinophil levels in the BAL fluid of DIO-OVA mice (S3 Fig.).

### 4. Treatments of obesity by exercise or normal diet reduce pulmonary inflammation in obesity-related asthma model

**Figure 5:**
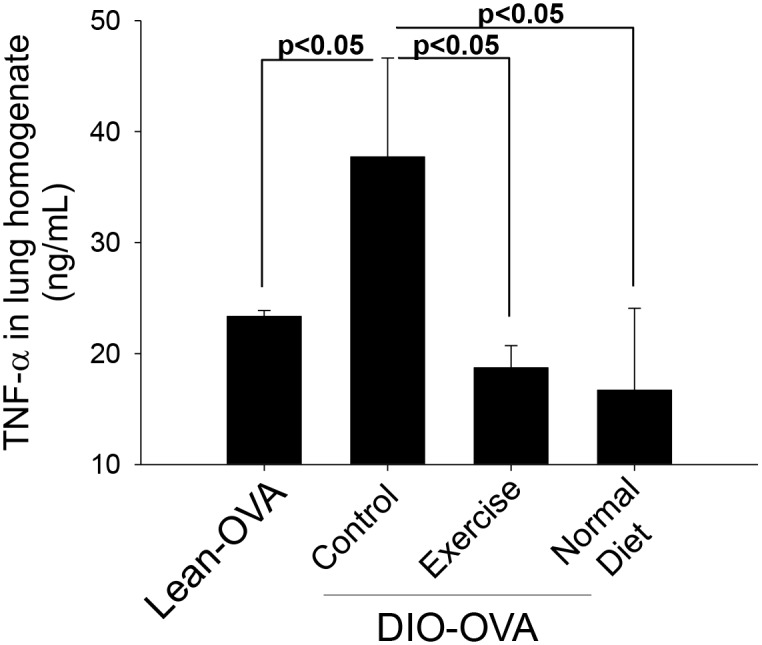
Treating obesity decreases TNF-α levels in the obesity-related asthma model. DIO mice performed voluntary exercise or consumed a normal chow diet to treat obesity. TNF-α levels in the lung homogenates were measured in the weight-reduced, obesity-related asthma mice. The solid lines indicate statistical significance between each group (p<0.05). Error bars indicated mean±SEM of five mice per group. All data are representative of three independent experiments.

**Figure 6:**
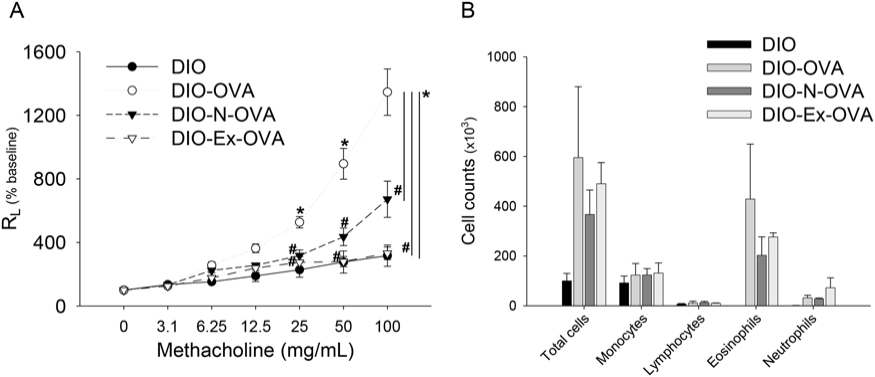
Treating obesity decreases lung dysfunction in the obesity-related asthma model. DIO mice performed voluntary exercise or consumed a normal chow diet to treat obesity. (a) Airway hyperresponsiveness and (b) inflammatory cell infiltration in the bronchoalveolar lavage fluid were measured in the weight-reduced, obesity-related asthma mice. *, Statistical significance to lean mice (p<0.05); ^#^, Statistical significance to DIO mice. *DIO-N-OVA*, DIO-OVA mice with diet-restriction; *DIO-Ex-OVA*, DIO-OVA mice with voluntary exercise. Error bars indicated mean±SEM of five mice per group. All data are representative of three independent experiments.

In this study, we identified that pre-existing obesity exacerbates subsequent asthmatic lesions via alveolar macrophages and their TNF-α production. For that reason, we investigated whether treating obesity ameliorates the severity of asthma. Both exercise and normal diet dramatically reduced TNF-α secretion from the lung homogenates of DIO-OVA mice (Fig. 5). However, leptin and adiponectin levels in the lungs and sera were not different among the groups (DIO-OVA, DIO-Ex-OVA and DIO-N-OVA; S4 Fig.). Both the DIO-Ex-OVA and DIO-N-OVA mice exhibited recovered AHR compared with the DIO-OVA mice. Surprisingly, the AHR of DIO-Ex-OVA mice improved to that of DIO mice (Fig. 6A). However, there were no significant differences in the inflammatory cell infiltrations (Fig. 6B), and total and specific IgE levels (data not shown) between the DIO-OVA and DIO-Ex-OVA groups.

## Discussion

Obesity is a disease of affluence and the prevalence of obesity has increased. Asthma, another non-communicable disease, also has increased. Some studies suggest that obesity plays a substantial role in the development, control, and severity of asthma [2, 18], and obesity-related asthma is one of the non-T_H_2 asthma phenotypes, lacking T_H_2 biomarkers and specific IgE [3]. In this study, we identified that airway resistance was exacerbated in obesity-related asthma mice. As exacerbated AHR was relieved by the restoration of normal body weight, we are convinced there is a relationship between obesity and asthma.

Many studies have supported an association between obesity and asthma, but the particular mechanisms for this linkage have not been fully investigated. As one possible cue, obesity is associated with a state of low-grade systemic inflammation and leads to an increased release of proinflammatory molecules that play a crucial role in the pathogenesis of obesity-associated complications [19, 20]. Because asthma is also an inflammatory disease, it is possible that obesity can worsen asthma symptoms and exacerbate asthma control. According to the Behavioral Risk Factor Surveillance System 2010 survey data and documentation, the obesity rate among adults with asthma is significantly higher than the rate among adults without asthma in the USA [21].

To clarify the relationship between obesity and asthma, adipose-derived secreted factors have been studied. Leptin, a typical adipokine, regulates feeding behavior through the central nervous system. It is produced in adipocytes and then secreted to the blood stream. Leptin levels in the blood positively correlate with adipose mass in human subjects, as well as in mice. Adiponectin has also been identified as an adipocyte-specific adipokine and its expression is found to be lower in obesity. In this study, leptin and adiponectin levels in both lung homogenates and blood sera were not noticeably different between the obese and obesity-treated mice. However, we found that TNF-α levels were greater in the lungs and blood of DIO-OVA mice. In clinical and epidemiological studies, TNF-α levels are greater in the adipose tissue and plasma of obese individuals, and weight control in these individuals is associated with a decrease in TNF-α expression [22, 23]. TNF-α can also exacerbate asthma and play a crucial role in the relationship between obesity and asthma [8].

Previously, we identified that TNF-α plays an important role in the etiology of asthma and alveolar macrophages can produce large amounts of TNF-α in the lung environment [15]. In this experiment, we confirmed that obesity prior to asthma could aggravate lung function and AHR, and systemic overexpression of TNF-α might be a typical mechanism to explain the exacerbated lung function. However, we could not find a significant difference between the DIO-OVA and lean OVA groups in their inflammatory cell profiles in BAL fluid, and this result may be due to our using an allergy OVA asthma model. Furthermore, some investigators have suggested that inflammation in adipose tissue and blood may be more important than in airways [13, 24]. They have shown that BAL fluid cytokine levels are greater after bariatric surgery.

In many studies, anti-TNF-α therapy has been used as a strategy for the treatment of asthma [9, 25] and this TNF-α blockade attenuates the production of proinflammatory cell infiltrations in BAL fluid, and airway remodeling and function in mouse models of asthma [15]. But a randomized clinical trial of anti-TNF-α treatment for severe asthmatics was terminated early because of serious drug side effects, such as pneumonia and malignancy [26]. Our study suggests that targeting obesity-associated asthma may be required for anti-TNF-α treatment in asthma.

Various types of immune cells that produce TNF-α exist in the lung environment and alveolar macrophages are one of these cell types. Activation of alveolar macrophages results in airway inflammation and pulmonary fibrosis [27, 28], and depletion of the alveolar macrophages may repress AHR and airway inflammation in allergic asthma [29, 30]. To confirm whether alveolar macrophages are the major source of pulmonary TNF-α production in obesity-related asthma mice, alveolar macrophage depletion using Cl_2_MDP-containing liposomes was performed. Our results reveal that the depletion decreased TNF-α levels in lung homogenates and recovered lung dysfunctions caused by asthma and obesity.

Judging from the relationship between obesity and asthma, it may be possible that an appropriate treatment of obesity leads to recovery of asthma lesions in the obesity-related asthma model. To investigate our last question, we proceeded to treating obesity.

Caloric restriction based on a low calorie intake, and additional energy expenditure by physical exercise, are typical treatments for obesity [31]. We examined the effects of a normal chow diet or voluntary exercise for body weight reduction. Several studies have suggested that forced exercise on treadmills induces undue stress [32], whereas voluntary exercise can reduce body weight without stress in obese mice [33, 34]. Actually, the obese mice in our study voluntarily ran 6 kilometers per day for 4 weeks (DIO-Ex), and both the DIO-Ex and obese mice with a normal chow dietary intake (DIO-N) significantly reduced their body weight.

Restoration of body weight certainly has a therapeutic effect on asthmatic diseases. In systematic reviews on obesity and asthma, several investigators have noted improvements in asthma outcome and control after weight loss in overweight or obese subjects [11–13, 35]. Similar to these preceding studies, we also observed that restoration of normal weight decreased TNF-α levels and ameliorated AHR in both DIO-Ex and DIO-N mice.

There are several missing points in this study that should be elucidated through further studies. Particularly warranted are investigations into which factors stimulate TNF-α-producing alveolar macrophages in the obesity-related asthma and which mechanisms are involved in the relationship between treatments of obesity and improved asthmatic lesions.

In summary, we identified that inflammatory response is a possible mechanism that can explain the relationship between obesity and asthma, and control of the inflammatory response through the TNF-α pathway is a means for treating obesity-related asthma. We also showed that restoring normal body weight reduces pulmonary TNF-α levels and then improves typical symptoms of asthma in obesity-related asthma mice, which suggests that restoring normal body weight is an appropriate treatment strategy for obesity-related asthma.

## Supporting Information

S1 ARRIVE Checklist (PDF)

S1 Fig Histological characteristics in the obese mice and obesity-related asthma mice. Lung tissues were stained with PAS. All data are representative of three independent experiments.
(TIF)

S2 Fig Depletion of TNF-α or alveolar macrophages decreases pulmonary function in the OVA asthma model. Lean-OVA mice was treated with TNF-α blockade antibody or Cl_2_MDP for depletion of TNF-α or alveolar macrophages, respectively. (a) Airway hyperresponsiveness and (b) alveolar macrophage levels in the lung tissue were measured in the TNF-α or alveolar macrophage depleted DIO-OVA. *, Statistical significance to lean mice (p<0.05); ^#^, Statistical significance to DIO mice. *TNF*, TNF-α neutralizing antibody. Error bars indicated mean±SEM of five mice per group. All data are representative of three independent experiments.
(TIF)

S3 Fig Depletion of TNF-α or alveolar macrophages changes immune cell phenotypes in the BAL fluid of the obesity-related asthma mice. DIO-OVA and Lean-OVA mice was treated with TNF-α blockade antibody or Cl_2_MDP for depletion of TNF-α or alveolar macrophages, respectively. Inflammatory cell infiltrations in the BAL fluid were measured in the TNF-α or alveolar macrophage depleted mice. Error bars indicated mean±SEM of five mice per group. All data are representative of three independent experiments.
(TIF)

S4 Fig Leptin and adiponectin levels in the obesity-related asthma and weight-reduced obese asthma model. DIO mice performed voluntary exercise or diet restriction for the treatment of obesity. (a) Leptin and (b) adiponectin levels of the lung homogenates and blood sera were measured in the weight-reduced obese asthma mice. *N.D.*, not detected. Error bars indicated mean±SEM of five mice per group. All data are representative of three independent experiments.
(TIF)
