# Supplementary figures and images for: Obesity Increases Airway Hyperresponsiveness via the TNF-α Pathway and Treating Obesity Induces Recovery
Source: PLoS One. 2015 Feb 6;10(2):e0116540. doi: 10.1371/journal.pone.0116540 (PMC4344461; doi:10.1371/journal.pone.0116540)

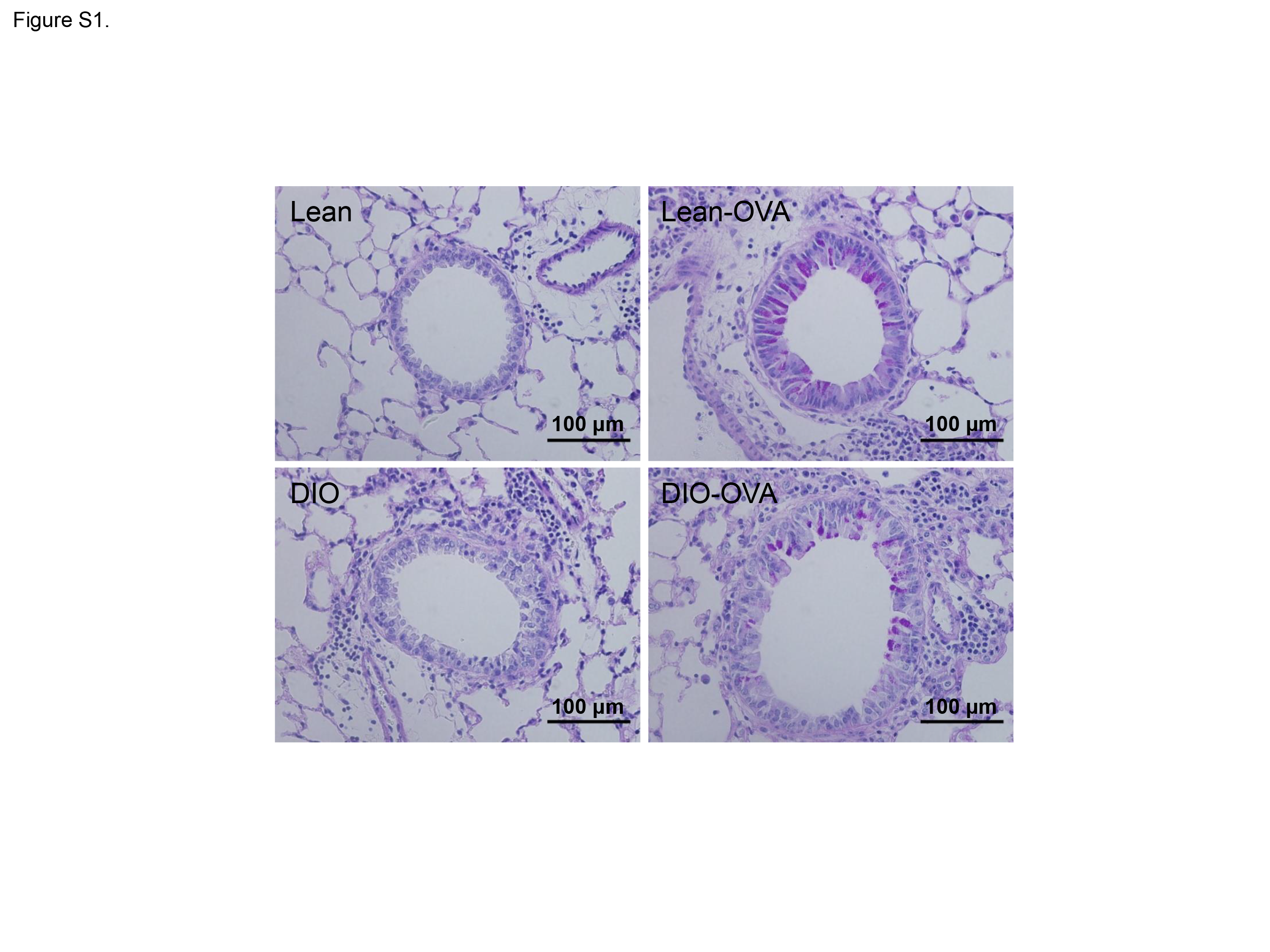

Supplement: S1 Fig — Lung tissues were stained with PAS. All data are representative of three independent experiments. (TIF) [file pone.0116540.s002.tif]

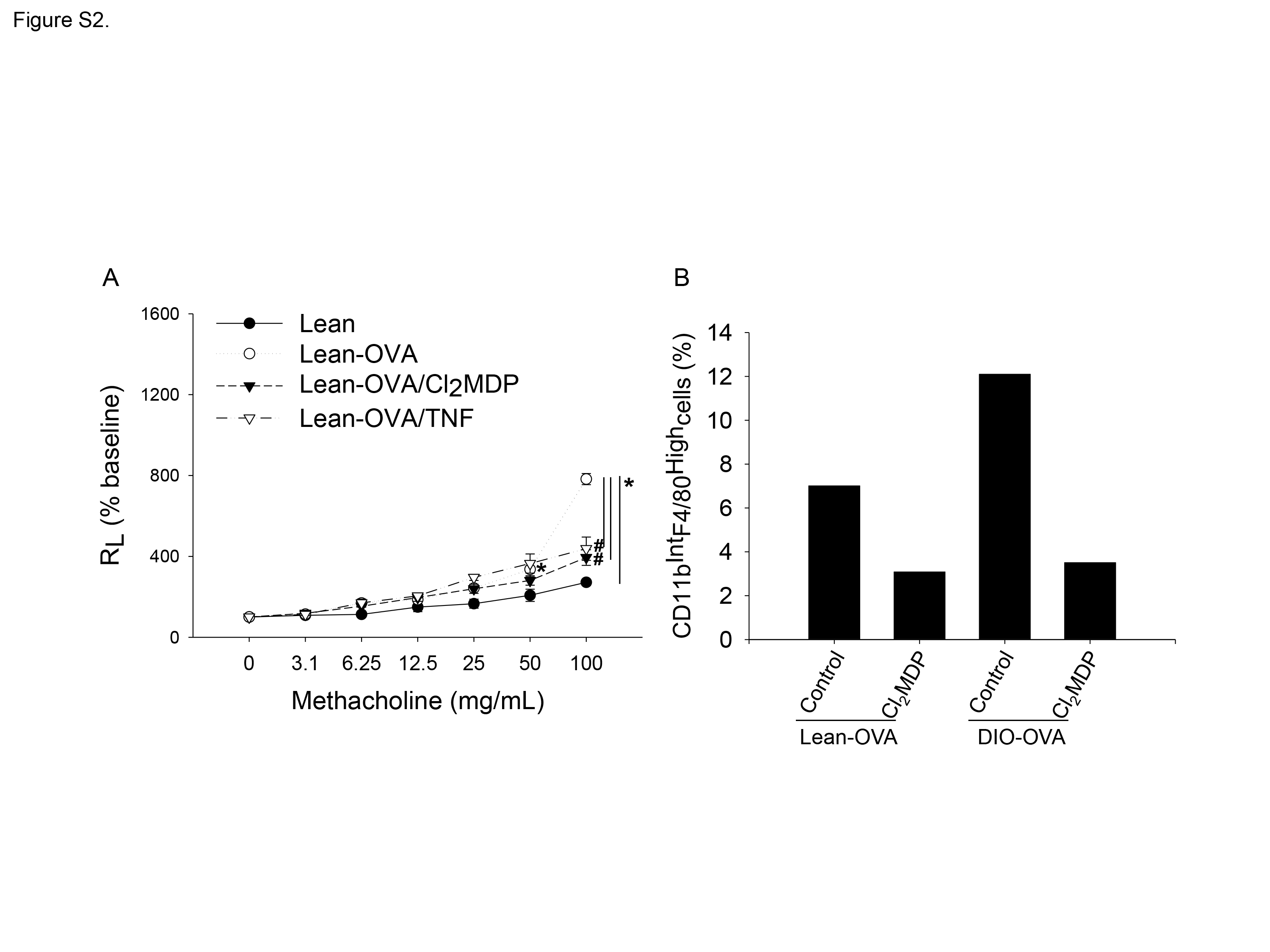

Supplement: S2 Fig — Lean-OVA mice was treated with TNF-α blockade antibody or Cl2MDP for depletion of TNF-α or alveolar macrophages, respectively. (a) Airway hyperresponsiveness and (b) alveolar macrophage levels in the lung tissue were measured in the TNF-α or alveolar macrophage depleted DIO-OVA. *, Statistical significance to lean mice (p<0.05); #, Statistical significance to DIO mice. TNF, TNF-α neutralizing antibody. Error bars indicated mean±SEM of five mice per group. All data are representative of three independent experiments. (TIF) [file pone.0116540.s003.tif]

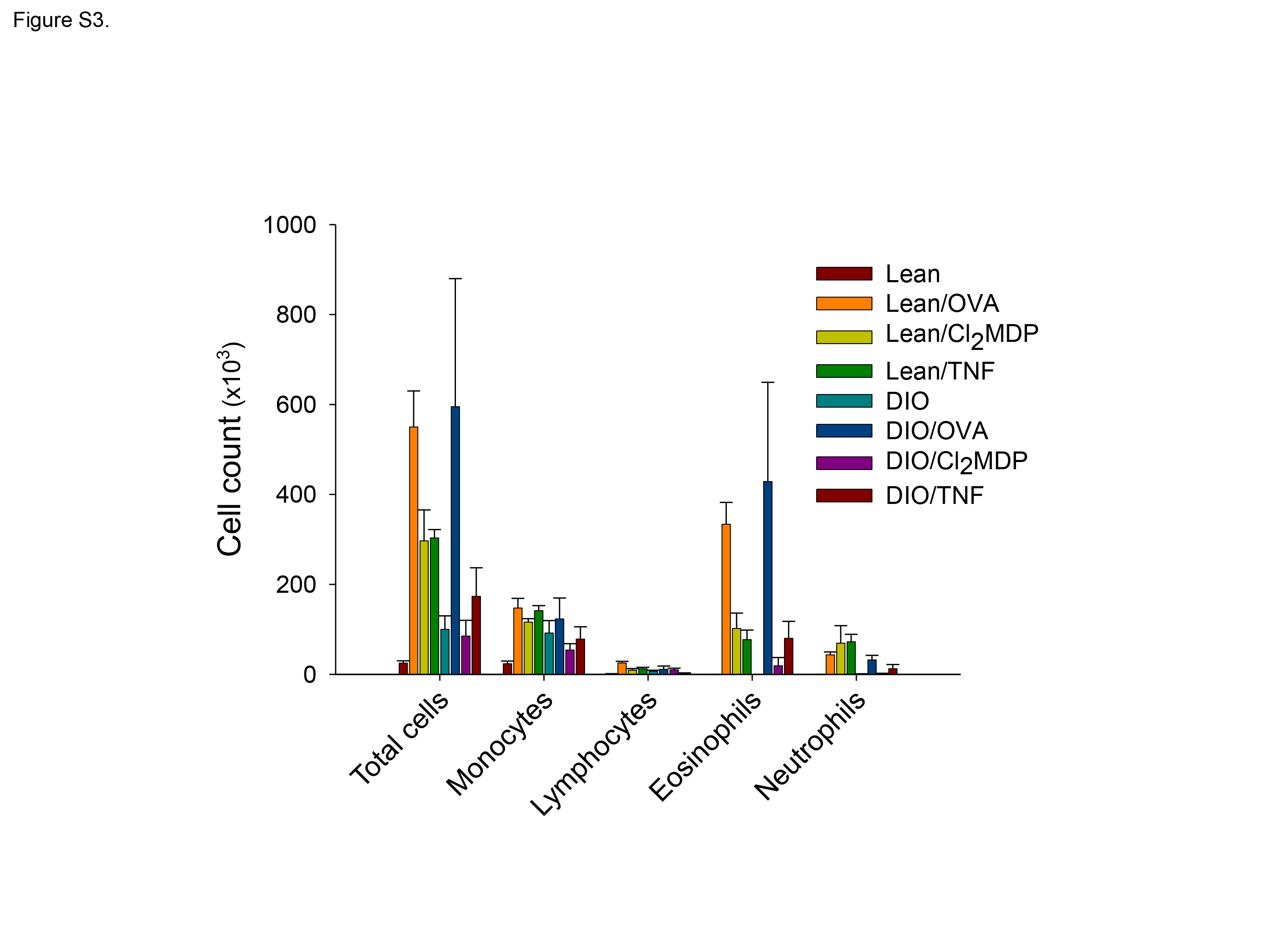

Supplement: S3 Fig — DIO-OVA and Lean-OVA mice was treated with TNF-α blockade antibody or Cl2MDP for depletion of TNF-α or alveolar macrophages, respectively. Inflammatory cell infiltrations in the BAL fluid were measured in the TNF-α or alveolar macrophage depleted mice. Error bars indicated mean±SEM of five mice per group. All data are representative of three independent experiments. (TIF) [file pone.0116540.s004.tif]

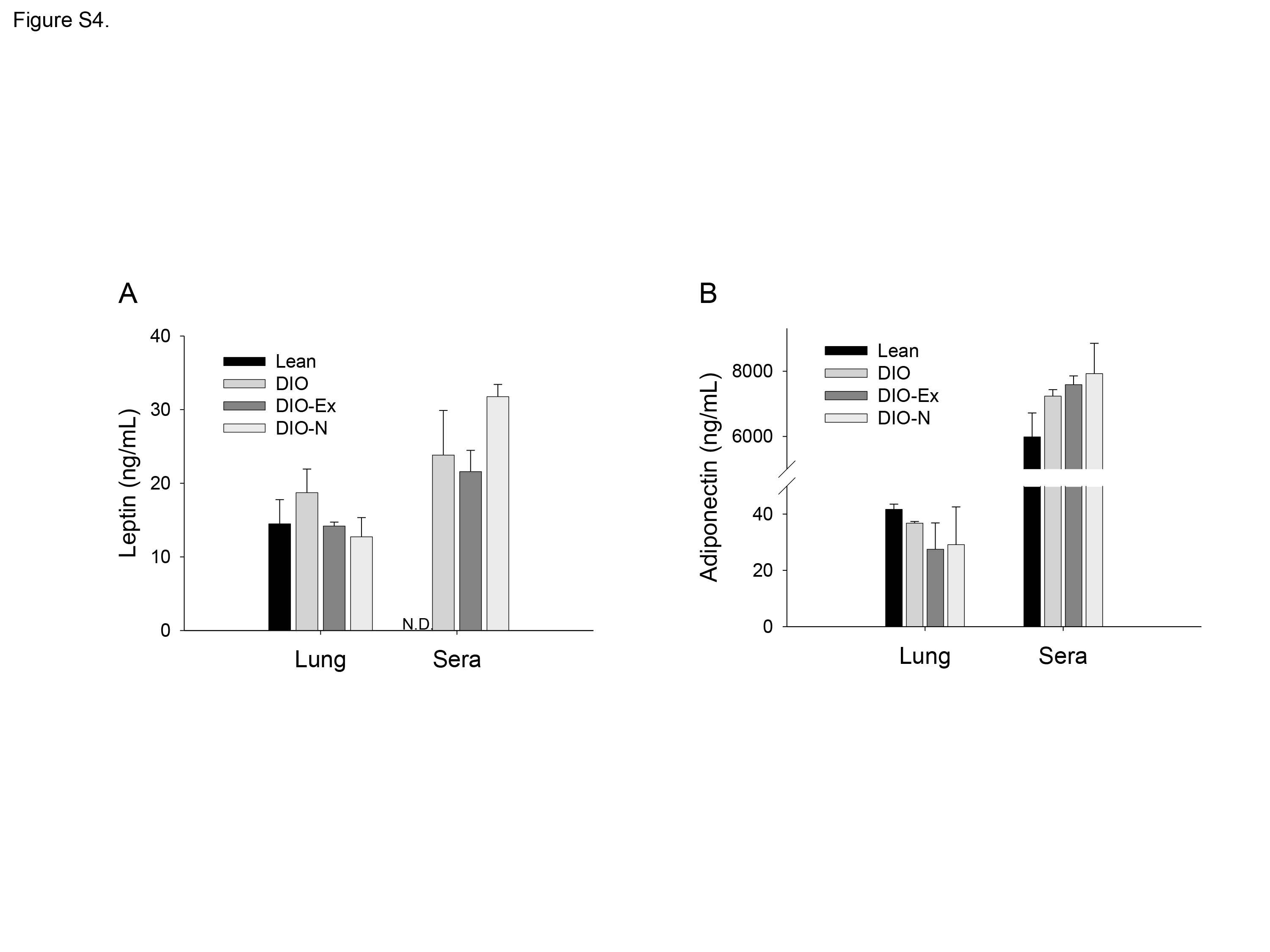

Supplement: S4 Fig — DIO mice performed voluntary exercise or diet restriction for the treatment of obesity. (a) Leptin and (b) adiponectin levels of the lung homogenates and blood sera were measured in the weight-reduced obese asthma mice. N.D., not detected. Error bars indicated mean±SEM of five mice per group. All data are representative of three independent experiments. (TIF) [file pone.0116540.s005.tif]
